# Designing integrated interventions to improve nutrition and WASH behaviors in Kenya

**DOI:** 10.1186/s40814-020-0555-x

**Published:** 2020-02-03

**Authors:** Kimberly R. Jacob Arriola, Anna Ellis, Amy Webb-Girard, Emily Awino Ogutu, Emilie McClintic, Bethany Caruso, Matthew C. Freeman

**Affiliations:** 1 Department of Behavioral Sciences and Health Education, Rollins School of Public Health, Emory University, Atlanta, GA USA; 2 Department of Environmental Health, Rollins School of Public Health, Emory University, Atlanta, GA USA; 3 Hubert Department of Global Health, Rollins School of Public Health, Emory University, Atlanta, GA USA

**Keywords:** Water, sanitation, and hygiene, Malnutrition, Child stunting, Behavioral theory, Behavior change intervention, Maternal and child health

## Abstract

**Background:**

Child stunting, an indicator of chronic malnutrition, is a global public health problem. Malnutrition during pregnancy and the first 2 years of life undermines the survival, growth, and development of children. Exposure to fecal pathogens vis-à-vis inadequate water, sanitation, and hygiene (WASH) has been implicated in the etiology of child stunting, highlighting the need to integrate WASH with nutrition-sensitive interventions to comprehensively address this complex problem. The aim of this study was to describe a systematic, theoretically informed approach (that drew from the Starr and Fornoff approach to the Theory of Change development and the Behavior Change Wheel approach) to design a multi-component and integrated social and behavior change intervention to improve WASH and nutrition-related behaviors in western Kenya.

**Methods:**

This intervention was developed to be integrated into an existing project that utilized the care group model and aimed to create a culture of care and support for HIV/AIDS-affected children under two and their caregivers and was executed by local partners. We tested the newly created intervention packages in user-testing trials using an adapted Trials of Improved Practices approach to pilot acceptability and feasibility.

**Results:**

Using authentic stakeholder engagement and relevant theories, we conducted an 8-step process: (1) conduct mixed methods formative research, (2) prioritize target behaviors, (3) use causal analysis to create problem trees, (4) develop solution trees and articulate assumptions and rationales for change, (5) link solution trees to intervention functions, (6) develop the intervention plan, (7) create the intervention packages, and (8) test and refine the intervention packages.

**Conclusions:**

This study highlights the need to take a multi-sectorial, integrated approach that integrates contextually relevant behavior change theories with the experiential knowledge gleaned from stakeholders into the design of interventions that seek to reduce child stunting. This process resulted in the creation of intervention packages that grouped behaviors thematically to be most relevant and responsive to the population context. This work has the potential to make important contributions towards achievement of the United Nations’ sustainable development goals.

## Background

Child stunting, an indicator of chronic malnutrition, is a global public health problem. Malnutrition during pregnancy and the first 2 years of life undermines the survival, growth, and development of children [1, 2]. The etiology of early childhood malnutrition is complex, involving interactions between parental feeding practices, dietary intakes, and nutrient absorptive capabilities. Gastrointestinal and other infectious diseases, determined by a household and community’s access to adequate water, sanitation, hygiene, and health services, increase nutritional needs and can further exacerbate malnutrition [3–5]. If not addressed, malnutrition can impair gross motor, fine motor, and cognitive development with later implications for schooling success and economic productivity in adulthood. Chronic malnutrition in early childhood also predisposes individuals to cardiovascular disease in adulthood [3–5]. Global leaders recently pledged to reduce chronic malnutrition among children under age five by 40% by 2025 [6]. Nutrition-specific interventions—those aimed at improving nutrient intakes and diet practices—are, on their own, insufficient to achieve these reductions even when delivered at scale and with high coverage [7, 8]. As a result, there is interest in developing, testing, and evaluating multi-sectorial and integrated approaches to tackle the underlying determinants of child nutrition [9, 10]. Known as nutrition-sensitive approaches, these include nutrition-sensitive agriculture [10, 11], poverty reduction [9], girls’ education [10, 12], birth spacing [13], and women’s empowerment [14].

In addition, research implicates early and chronic, even asymptomatic, infections with fecal pathogens in the etiology of stunting [15, 16]. Clean drinking water, sanitation facilities that hygienically separate feces from human contact, and appropriate hygiene behaviors, such as handwashing with soap—together known as WASH—can reduce exposures to harmful enteric pathogens of both human and animal origins [17]. In the absence of these environmental improvements and associated behaviors, children are at risk of stunting due to extended and repeated occurrences of diarrhea [18], soil-transmitted helminth infections [19], and asymptomatic infections that itself can lead to environmental enteric dysfunction, a chronic condition that reduces intestinal nutrient absorption [20, 21].

However, field trials have found limited efficacy and effectiveness of improving WASH on child growth [22–24]. While it may be that improvements to the hygienic environment do not impact stunting, it is more likely that they did not sufficiently reduce exposure to fecal pathogens because the interventions were not appropriate, were not delivered optimally, or did not sufficiently target the appropriate exposure pathways [25, 26].

One critical limitation of many studies assessing the impacts of WASH and nutrition interventions is that the approaches to change behavior are delivered in parallel, rather than as integrated packages. Menon and Frongillo [26], in their recent commentary, highlighted the need for integrated approaches that target the caregiving dyad’s lived reality and the social (i.e., household and community members) and physical environment within which caregiving occurs. They recommended including or perhaps especially, integrating behavior change efforts seamlessly into daily routines. Moreover, the theoretical foundations of WASH and nutrition interventions are rarely described, and thus, the pathways explaining how interventions are expected to change behavior and ultimately reduce child stunting are poorly articulated [27–31]. Recent reviews highlight a need to provide sufficiently detailed descriptions of the process, theory, and evidence bases used to develop stunting reduction programs and their theories of change [29, 30, 32–34]. Such detail fosters transparency, allows for replication, and gives greater insights into what specific techniques work to change behavior and why.

## Theoretical and methodological foundations

To that end, this study sought to develop an integrated WASH/nutrition behavior change intervention. To design the intervention, we applied two relevant theoretical and methodological frameworks: (1) the Starr and Fornoff [35] approach to Theory of change (TOC) development and (2) the Behavior Change Wheel (BCW) approach [36] to intervention design.

Theories of change are widely used in designing, planning, managing, evaluating, and scaling complex interventions [27, 34, 37, 38]. They are often developed through a backwards mapping approach that starts with a clear goal statement and then maps the required process of change to achieve that goal through delineation of antecedent outcomes, behaviors, and behavioral determinants [38]. This sequence of change is developed with a deep understanding of context, including the assumptions, motivations, worldviews, and philosophies of relevant stakeholders [38]. In addition to the sequence of change, TOCs articulate assumptions related to the reach, coverage, capacity change, program fidelity, behavior change, and benefits [39]. Such delineation permits a robust examination of the process of change. Experts recommend that a TOC should be developed as part of a multi-stakeholder and collaborative experiential learning exercise [40]. The Starr and Fornoff [35] process for TOC development entails engaging stakeholders to describe a current problem through formative research, map the underlying causes, identify the long-term changes needed to address the problem, and develop the activities that need to occur to create the desired long-term change.

Given that a TOC is a pragmatic framework for describing *how* an intervention will produce change, it is strengthened by the integration of key behavioral, social, or psychological theories that explain *why* certain pathways to change are expected to exist [27, 28]. If integrated into a TOC properly, these theories explain the types of behavior change that are expected to occur and aid in specification of activities that may produce the desired behavior change. To that end, we adapted key steps from the BCW approach [36] to develop the intervention plan.

The BCW approach characterizes behavioral systems using the “COM-B” model. Analogous to that of Rothschild [41], the COM-B model hypothesizes that volitional behavior is determined by behavioral *capability* (or the physical and psychological skills necessary to perform the behavior), *opportunity* (or the physical and social supports or restraints on the behavior), and *motivation* (or the reflective and automatic processes that direct behavior). The BCW process, like the Starr and Fornoff approach to TOC development, begins with understanding and defining priority behaviors in a given context using formative research. Following specification of the behavior (i.e., the who, what, when, where, how often, and with whom) and using findings from formative research, the BCW approach applies the COM-B model to identify and characterize specific behaviors and behavioral antecedents known as theoretical domains [42]. These domains bridge to relevant behavior change approaches referred to as intervention functions [36, 42]. A taxonomy of behavior change techniques [43, 44] is then used to map intervention functions to specific and appropriate approaches that facilitate behavior change.

## Aims

Aligned with a call to document intervention development processes, our manuscript describes a systematic and theoretically informed approach to design an integrated social and behavior change intervention to improve WASH and nutrition-related behaviors in western Kenya, with the goal of reducing child stunting [45]. Through application of the BCW to this work, we developed three thematic intervention packages to reduce child stunting: (1) food preparation and storage, (2) mealtime behaviors, and (3) clean family environment (see Table 1) [36]. Our approach balanced evidence from the peer-reviewed literature and stakeholder expertise. Our goals in presenting this detailed description of our intervention design process include fostering transparency, enabling replication, and providing greater insights into what specific techniques work to change behavior and why.

**Table 1:** Final list of targeted nutrition and WASH behaviors

Behavioral category	Behavior
1. Food preparation and storage	1a. Handwashing with soap before food preparation • Caregiver washes hands with soap before food preparation 1b. Food safety during preparation • Food is washed and raw meat is separated from other ingredients • Food is fully cooked • Leftover food is reheated after 4 h of initial cooking • Utensils are fully cleaned and dried 1c. Food storage • Stored food is covered • Stored food is kept in clean container
2. Mealtime behaviors	2a. Improved dietary diversity • Improved dietary diversity with locally available foods for pregnant and lactating women • Improved dietary diversity with locally available foods for children under 2 using locally available food • Improved dietary diversity with locally available food for whole family throughout the life course 2b. Children under 2 and pregnant and lactating women are given adequate food • Children under 2 are fed complementary foods of appropriate thickness • Children under 2 are fed complementary foods with appropriate frequency • Children under 2 are fed complementary foods in appropriate portions • Child under 2 takes extra meal/snack • Pregnant and lactating women take extra meal/snack 2c. Feeding based on child-demonstrated hunger • Caregiver recognizes cues to hunger before child 0–12 months begins crying (putting fingers in mouth, spits, looking at others eating) • Child 6–24 months is fed slowly and patiently, using eye contact, encouraging and motivating the child to eat 2d. Handwashing with soap before feeding and eating • Caregiver washes hands with soap before eating • Caregiver washes hands with soap before feeding CU2 • Caregiver washes child’s hands with soap before feeding/eating
3. Clean family and home environment	3a. Hygienic play environment for children under 2 years of age • Rapidly dispose of animal feces in latrine • Sweeping/cleaning of compound 2–3 times per day 3b. Safe disposal of child feces • Caregiver rapidly disposes child feces in latrine 3c. Handwashing with soap after child feces disposal • Caregiver washes hands with soap after disposing of child feces • Caregiver washes child's hands after defecation 3d. Handwashing with soap after defecation • Caregiver washes hands with soap after defecation 3e. Nails are clipped on regular basis • Caretaker clips child’s hands so fingernails do not extend over fingertips

## Methods

The integrated WASH/nutrition behavior change intervention was designed to be implemented as part of the THRIVE II project executed by local partners of Catholic Relief Services, the primary implementing partner, in Kenya. THRIVE II was a two-year project that began in January 2016, of which the goal was to create a culture of care and support for HIV- and AIDS-affected children under 2 years and their caregivers in Kenya, Tanzania, and Malawi. It represented work that continued from the initial project, THRIVE, which had the same overall goal. The local partners, Homa Hills Community Development Organization and Mercy Orphans, were responsible for the supervision and ground support of the project. Using the Care Group model [46], THRIVE II provided ongoing support to caregivers of children under two years of age to practice early childhood stimulation, positive parenting, and optimal infant and young child feeding and WASH behaviors. Community health workers trained volunteer lead mothers who disseminate health messaging to small groups (6–12 mothers) and conduct home visits to support ongoing behavior change. At the community level, strategies were implemented to strengthen the capacity of health facilities to support early stimulation and positive parenting counseling. In Kenya, the project targeted an estimated population of 3120 pregnant mothers and caregivers of children below 2 years of age.

We tested the newly created intervention packages in user testing trials using an adapted Trials of improved practices (TIPs) approach [47]. User testing sought to give participants voice in program design and to gauge the acceptability and feasibility of the intervention components as a way to maximize uptake and effectiveness. The duration of TIPs varies depending on the behavior but often lasts 5–7 days for nutrition-related iterations. Trials of improved practice often occur over three visits, with the first visit used to assess challenges performing the optimal behaviors, the second visit to provide feedback and negotiate adoption of potential solutions, and a third visit to evaluate uptake and gather family perspectives on the solution. Analysis of both quantitative and qualitative data from these three visits serves to identify key determinants of and feasible solutions for problem behaviors. Sample size is typically 20–50 families, selected for heterogeneity [47]. We adapted the traditional TIPs process by focusing our analysis on the feasibility and acceptability of the newly developed intervention packages and extended the user testing period beyond the usual 5–7 days.

We held to the following key foundational principles throughout the intervention design process: (1) Authentic stakeholder engagement is crucial to the successful design of the intervention, (2) relevant theories of behavior change should inform the design and development of the intervention, (3) the intervention should target individual and/or community-level behavior (versus policy or physical structures), (4) the intervention must be able to be delivered by trained community health volunteers in the context of the existing intervention (Care Group model), and (5) the intervention must have the potential for sustainability beyond the life of the project.

## Results

### Step 1: conduct mixed methods formative research

The first step in our process, formative research, informed “how best to incorporate aspects of programme design and implementation into the environmental and cultural context” [48–50], (p., 64). This step served to identify the suite of relevant behaviors for action, build the evidence to support pathways of change, and identify existing resources that could support positive behavior change [35]. We conducted formative research in two phases: desk review and primary data collection. We first reviewed the findings of mixed methods research conducted by the THRIVE II program in the program areas of Homa Bay County and Migori County [51]. The desk review served to identify (1) optimal WASH and maternal and child nutrition behaviors with low uptake, (2) the socio-demographic characteristics of the communities, and (3) gaps in understanding key habits, knowledge, beliefs and attitudes, barriers and facilitators to optimal maternal/child nutrition, hygiene, and sanitation behaviors [51]. Findings from the desk review informed the protocols and guiding questions for a second round of ethnographic research that aimed to provide greater contextual detail, clarify key barriers and facilitators of behavior change to infant and young child feeding and WASH-related behaviors, identify messages that resonated with caregivers, explore in more depth the opinions and experiences of caregivers, and determine potential sources of information [52]. Data were collected in six rural communities in Migori (*n* = 3) and Homa Bay Counties (*n* = 3). Data collection consisted of market surveys (*n* = 4); focus group discussions with pregnant women and mothers of children under two, fathers, and grandmothers (*n* = 24); key informant interviews with community health workers, community and religious leaders, implementing partners, and staff at the funding agency (*n* = 29); household spot-checks to ascertain WASH hardware and general hygiene (*n* = 12); and structured household observations of WASH and feeding behaviors (*n* = 24). Based on a total of 83 activities that reached 288 study participants, the results indicated a range of barriers and facilitators that influenced families’ practice of maternal and child nutrition and WASH behaviors (Table 2). These findings are elaborated elsewhere [52].

**Table 2:** Summary of drivers and barriers to key IYCF and WASH behaviors based on formative research

Key behavior	Barriers	Drivers
Infant and young child feeding	▪ Belief that eating specific food when pregnant will result in a too large baby (*maternal nutrition)* ▪ Lack of time to breastfeed, prepare complementary foods multiple times per day, practice responsive feeding ▪ Belief that covering hot food will degrade quality	▪ Caregiver and family member awareness of critical foods during pregnancy and lactation (*maternal nutrition.* ▪ Knowledge of breastfeeding benefits for CU2 (*EBF*) ▪ Access to and knowledge of drying racks through community strategy (*food hygiene*)
Household water treatment	▪ Limited access to water ▪ Unacceptability of chemical treatment taste and smell ▪ Access to chemicals inconsistent at health facilities; cost barrier if purchased outside of health facility ▪ Perceived lack of time to collect firewood to boil water	▪ Knowledge of multiple water treatment techniques, including: adding alum, boiling, straining, letting water settle, and treatment with chemicals (PUR & Waterguard). ▪ Perceived importance of cleaning water storage containers
Handwashing with soap at critical times	▪ Limited access to water and soap. ▪ Handwashing with soap is not a perceived social norm. ▪ Concern that soap or water at handwashing station will be consumed by animals, stolen, ruined/damaged by children ▪ Perceived lack of time to fill handwashing stations daily	▪ Convenience of handwashing station (handwashing station near latrine, access to soap) ▪ Disgust of feces or dirt on hands ▪ Caregiver knowledge of when to wash own hands
Latrine use	▪ Lack of household latrines ▪ Latrine building challenge because of soil, affordability of materials, and limited skilled workers ▪ Public urination and defecation is socially acceptable. ▪ Low acceptability of latrines due to smell, cleanliness, safety, ownership, and distance from compound	▪ Privacy during urination and defecation, particularly of women ▪ Disgust related to the sight and smell of feces Perceived fear of catching diseases (e.g. typhoid, cholera)
Safe child feces disposal	▪ Lack of household latrines ▪ Lower perceived risk of disease associated with child feces ▪ Perceived lack of time for caretakers to supervise children (do not know where/when child defecates)	▪ Disgust related to the sight and smell of feces, presence of flies associated with feces in the compound ▪ Caretakers train children to defecate in designated location
Promoting clean play environment	▪ Uncontained compound animals result in presence of animal feces ▪ Lack of commonly understood definition for “protected play environment” ▪ Social acceptability of children freely playing around the compound, uncontained	▪ Social norm of child playing under caregiver supervision ▪ Social norms of keeping a clean compound and the habit of sweeping driven by feelings of disgust
Deworming	▪ Inconsistent information about dose frequency being given to caregivers by health workers ▪ Belief that the costs outweigh the benefits of taking the medication ▪ Religion forbidding the use of medication	▪ Caregivers perceived outcomes as positive for people that took de-worming medication. ▪ Knowledge is spread to community by community health volunteers and community health facility workers about de-worming medication.

### Step 2: prioritize target behaviors

Step 2 served to focus the scope of the intervention by prioritizing target behaviors. Informed by the formative research, we prioritized a preliminary set of nine target behaviors for intervention development: optimal maternal diet, exclusive breastfeeding, optimal complementary feeding, maternal handwashing, washing of children’s hands, safe child feces disposal, latrine use, handwashing station presence and use, and food hygiene. Four criteria informed this selection: (1) The behavior was not widely practiced, (2) there were opportunities to integrate behavior-specific activities into current THRIVE II programming, (3) there was plausibility or evidence of the relationship between the behavior and child stunting based on scientific research, and (4) the potential for changing the behavior was plausible given available resources and the scope of THRIVE II activities. This preliminary list of behaviors evolved over subsequent steps as stakeholders offered continued input, illustrating the iterative nature of this process.

### Step 3: use causal analysis to create problem trees

The aims of step 3 were to clearly articulate problem statements (e.g., handwashing at key times with soap is not practiced), identify strong and weak causal linkages for each of the identified problem behaviors, and create visual diagrams that specified the hierarchy of problems and causes, and cross-causal linkages [35]. We used problem trees to visualize the root and interrelated causes of target problem behaviors for stakeholder engagement. Problem trees are widely used in participatory approaches to develop health promotion interventions in developing countries [53]. The study team used findings from step 1 and the initial list of behaviors identified in step 2 to generate preliminary problem trees for each of the target behaviors, articulating causes at multiple levels (e.g., household, community). The problem trees were then refined during an intensive 3-day workshop with stakeholders, including government workers, local implementing partners, technical experts, and staff members from the NGO central and local office in Kisumu, Kenya.

### Step 4: develop solution trees and articulate assumptions and rationales for change

Following the production of problem trees, stakeholder workshop participants collectively drafted solution trees for each priority behavior, which corresponded to the problem trees. Similar to processes in Intervention Mapping [54, 55], solution trees translate the behavioral problems identified in problem trees (i.e., early introduction of non-breastmilk substances) into positive goal statements (i.e., refrains from giving liquids/foods before 6 months of age) [35]. Using a backwards mapping approach [27, 56, 57], positive actions, cognitive processes, and behaviors needed to achieve each of the priority behaviors were mapped. In converting problem trees to solution trees, we recognized the need for greater specificity in how we articulated the problems. For example, in conceptualizing solutions to achieve handwashing goals, we noted that “key times” for handwashing mattered and thus required narrower categories and specification (e.g., “caretakers wash hands with soap before eating” or “caretakers wash hands with soap before preparing food”).

Stakeholders reviewed solution trees during the 3-day workshop, identified needed revisions, and voted on priority behavioral determinants based on feasibility within the programming context and potential for change. Additional behavioral determinants linked to the potential for impact on stunting were identified during the post-workshop revision process. Once completed, each solution tree included multiple, linked levels of motivational, cognitive, and behavioral solutions phrased as goal statements (e.g., caregivers are motivated to wash hands; caregivers have access to soap) that if achieved would theoretically result in the practice of the priority behavior (e.g., caretakers wash hands with soap before eating). A final, collective review of the solution trees and goal statements eliminated those that would be outside of the scope of the project due to budget, time, existing programming, staff expertise, or those that did not align with the aims of creating a behavioral intervention (e.g., a policy-level intervention or a major structural intervention altering the environment).

### Step 5: link the solution trees to intervention functions

Step 5 served to verify the hypothesized pathways of change articulated in the solution trees and to link goal statements, COM-B components, theoretical domains, and intervention functions [36]. Two team members independently mapped each goal statement (e.g., caretakers wash hands with soap before eating) with behavioral determinants from the solution trees (e.g., washing hands before food preparation is perceived as convenient) onto one of the six corresponding COM-B components (e.g., reflective motivation). Mapping outputs were compared and assessed for consistency across researchers.

Next, we applied the theoretical domain framework (TDF) to each of the goal statements and the determinants identified in the solution trees [36]. The TDF, developed and validated through systematic review and consensus analysis, includes 128 explanatory constructs from 33 behavior change theories [58, 59]. It serves as an integrative framework synthesizing important constructs across many relevant theories, comprising 14 domains, including knowledge, skills, beliefs about capabilities, reinforcement, etc. The COM-B components served as a “cross-walk” linking the goal statements and their determinants to the TDFs. After mapping the domains to the goal statements and determinants, we identified specific intervention functions (e.g., education, persuasion, modeling) that corresponded with the relevant TDF. The culminating product of this process was an intervention function table (Table 3). When shared with Catholic Relief Services, they encouraged the inclusion of more behaviors that they felt would be manageable and critical given the local context. This negotiation ended with the addition of behaviors that would pointedly address environmental hygiene in the compound, as well as expanding upon handwashing and food hygiene behaviors (Table 1). Additional problem and solution trees were then developed, and step 5 was repeated for these new behaviors [58, 59].

**Table 3:** Intervention function table

Determinant(s) (COM-B)	Theoretical domains framework	Intervention function	Behavior change techniques	Intervention activity	Desired outcome
Section A: “clean compound” package					
Physical opportunity	Environmental context and resources	Environmental restructuring	Adding object to the environment	Feces scooper	Reduced instance of observed feces in the compound/in child play area
Reflective motivation	Intentions/belief about capabilities	Enablement	Behavioral contract, action planning	Pledge card	Households improve or adopt key clean compound behaviors selected during the initial counseling session.
Physical opportunity	Environmental context and resources	Environmental restructuring/training	Adding objects to the environment (demonstration of the behavior, rehearsal/practice of the behavior)	Nail clippers	Reduced child exposure to harmful pathogens transmitted by way of the fecal–oral route
Automatic motivation	Emotion	Modeling	Incompatible beliefs, information about health consequences	Clean compound family storybook	Reduced instance of observed feces in the compound/in child play area
Reflective motivation	(Social/professional role and identity)	Persuasion			
Physical opportunity automatic motivation	Environmental context and resources	Environmental restructuring	Prompts/cues, restructuring the physical environment	Handwashing station with soap delivery	Increased caregiver handwashing with soap after latrine use and after child feces disposal; increased handwashing with soap of CU2 after defecation
Section B: “mealtime” package					
Psychological capability	Behavioral regulation	Enablement	Action planning, self-monitoring	Dietary diversity tracking card	Increased consumption of diverse and appropriate hygienic complementary foods for children under 2; increased consumption of diverse foods for whole family
Reflective motivation	Intentions/belief about capabilities	Enablement	Behavioral contract, action planning	Pledge card	Households improve or adopt key mealtime behaviors selected during the initial counseling session.
Psychological capability	Skills	Training	Instructions on how to perform a behavior/ demonstration of the behavior	Feeding bowl counseling card	Increased consumption of diverse and appropriate, hygienic complementary foods for children under 2; increased consumption of diverse foods for pregnant and lactating women; increased caregiver use of responsive feeding techniques
Physical opportunity	Environmental context and resources	Environmental restructuring	Adding objects to the environment, prompts/cues	Feeding bowl and slotted spoon	Increased consumption of diverse and appropriate, hygienic complementary foods for children under 2; increased consumption of diverse foods for pregnant and lactating women; increased caregiver use of responsive feeding techniques
Psychological capability	Skills	Training	Behavioral practice/rehearsal, demonstration of the behavior	Community cooking/feeding demonstration	Increased consumption of diverse and appropriate, hygienic complementary foods for children under 2; increased consumption of diverse foods for pregnant and lactating women; increased caregiver use of responsive feeding techniques
Section C: “food hygiene” package					
Physical opportunity	Environmental context and resources	Environmental restructuring	Restructuring the physical environment	Handwashing station with soap delivery	Increased caregiver handwashing with soap and handwashing of children under 2’s hands before feeding and eating
Automatic motivation	Reinforcement	Environmental restructuring	Prompts/cues		
Psychological capability	Memory, attention and decision processes	Education/training	Prompts/cues, demonstration of the behavior	Food hygiene graphic card	Increased caregiver handwashing with soap before food preparation; reduced contamination of household foods
Reflective motivation	Intentions/belief about capabilities	Enablement	Behavioral contract, action planning	Pledge card	Households improve or adopt key food hygiene behaviors selected during the initial counseling session.
Physical opportunity	Environmental context and resources	Environmental restructuring	Restructuring the physical environment	Handwashing station with soap delivery	Increased caregiver handwashing with soap before food preparation
Automatic motivation	Reinforcement		Prompts/cues		
Physical opportunity	Environmental context and resources	Environmental restructuring	Adding object to the environment	Mesh food cover	Reduced fecal contamination of household foods
Social opportunity	Social influences (social norms)	Interpersonal influences, cultural expectations	Restructure the social environment	Roles and responsibilities/maize exercise	Increased caregiver recognition of potential inputs to improve children’s health
Reflective motivation	Social role	Education; Modeling			
Automatic motivation	Emotion	Modeling	Demonstration		
Reflective motivation	Intentions/belief about capabilities; social role	Enablement; Modeling	Behavioral contract, Action planning; goal setting	Public pledge with peer group	Households improve or adopt key food hygiene behaviors selected during the initial counseling session
Capability (physical and psychological)	Knowledge, physical skills	Education; training	Demonstration; instruction	Feeding demonstration	Increased caregiver knowledge of optimal porridge thickness and potential porridge additions; increased acceptability of porridge thickness;
Reflective motivation	Beliefs about consequences, optimism	Education; modeling	Feedback on outcome of behavior		Increased caregiver understanding that children could eat thick porridge without choking
Psychological capability	Knowledge	Education,	Information on health consequences,	Skits delivering intervention messages	Increased caregiver knowledge of ideal WASH/IYCF behaviors associated with their intervention package
Reflective motivation	Social role	Modeling	Demonstration of the behavior		
Physical opportunity	Environmental context and resources	Environmental restructuring	Restructuring the physical environment	Hardware demonstrations	Increased caregiver use of intervention hardware as intended
Capability (physical and psychological)	Knowledge	Education; training	Prompts/cues		
Psychological capability	Knowledge	Education; training	Demonstration; instruction	Household counseling	Increased capacity and motivation of caregivers to practice ideal WASH/IYCF behaviors
Social opportunity	Social Influence	Modeling, enablement	Social support; goal setting; problem solving; reviewing behavioral goals		

** Michie, van Stralen, West [44]^,^Michie, Richardson, Johnston, Abraham, Francis, Hardeman, Eccles, Cane, Wood [59]

### Step 6: develop the intervention plan

The aim of step 6 was to identify potential behavior change techniques that would utilize the intervention functions to reach our desired outcomes. The use of the overall delivery modality—the THRIVE II Care Group model—was pre-specified as part of the overarching intervention plan. Beyond this approach, we could develop new social behavior change communication material targeting community stakeholders (e.g., fathers). We narrowed potential intervention approaches and outputs by triangulating information from steps 1 to 5. Next, we identified the behavior change techniques that corresponded with the intervention functions selected in step 5. Behavior change techniques can be described as “observable, replicable, and irreducible component of an intervention designed to alter or redirect causal processes that regulate behavior” [60]. We reviewed the literature on WASH and nutrition-sensitive behavior change projects with similar desired outcomes. We analyzed elements of these interventions by applying the same taxonomy and steps (described in step 5) to the intervention components to understand what had been effective in other locations and which behavior change techniques had potentially informed activities within successful interventions [22, 60–65].

### Step 7: determine the intervention activities

The objective of this step was to develop specific intervention activities and package them thematically. We developed intervention tables to show the connection between the positive health goal statement from our solution trees with COM-B domains, the TDF, intervention functions, and behavior change techniques. In determining what the intervention activities would be, there was a need to address the challenge of impacting multiple behaviors within the limited timeframe of TIPs without reducing participants’ capability and motivation to practice desired behaviors. Since the behaviors were linked to daily household activities, we grouped them into three separate thematic packages. The “clean compound” package (Table 3, Section A) aimed to improve compound environmental hygiene to reduce children’s exposure to animal and human feces. The “mealtime” package (Table 3, Section B) aimed to improve caretaker behaviors that related to household eating and feeding times. The “food hygiene” package (Table 3, Section C) aimed to improve caretaker behaviors related to safe food preparation and storage.

After grouping behaviors together, we assessed which behavior change techniques were most appropriate for each behavioral outcome, what potential activities would come from behavior change techniques and how they could be adapted within the Care Group model. For example, the COM-B domain of social opportunity was addressed by incorporating grandmothers and fathers into community and household activities. The behavior change technique of social opportunity was approached through activities encouraging lead mothers to model these behaviors in their community.

### Step 8: test and refine intervention packages

We adapted TIPs to test and refine the intervention packages in the context of the Care Group model. Six neighbor groups from Homa Bay (*N* = 3) and Migori (*N* = 3) counties were evenly distributed to one of the three intervention packages. Fathers and grandmothers were included in community and household events. Trials of improved practices were implemented over 5 weeks. Trained research assistants, community health workers, and family members participated in a community event to (1) raise awareness of the assigned intervention package, (2) explore family members’ roles and contributions to child health, (3) share knowledge on targeted practices of their intervention arm (see Table 3), and (4) make a public pledge of commitment to improve practices. Research assistants conducted three household visits. First, research assistants documented current behaviors, introduced intervention materials, targeted behaviors for change, discussed tailored strategies for behavior change, and recorded participant pledges to practice behavior. After 3 weeks, research assistants monitored use of materials, problem-solved challenges, addressed knowledge gaps, and encouraged participants to maintain behaviors. At 5 weeks, researchers collected participant feedback on the intervention package including delivery, materials, strengths, and weaknesses; recorded participant uptake of key messages; and documented behavior change. Focus group discussions with mothers (*N* = 6) assessed participant acceptability of messages and materials, and family participation and engagement in the promoted practices.

Findings from TIPs demonstrated that thematic packaging of behavior change strategies assisted people in adopting more than one behavior. Some strategies necessitated change: we emphasized social opportunity in TIPs to overcome specific physical opportunity barriers (e.g., time, money, access to economic opportunities). To achieve this, we included fathers and grandmothers in activities and utilized social norms, pressures, and people’s roles towards their child(ren) to encourage all caregivers to change behaviors. However, fathers and grandmothers found it difficult to attend household counseling sessions. Mothers were influenced by other women, whether neighbors, co-wives, or community health workers.

We adapted the TIPs approach to accommodate the Care Group model and emphasize the social relations of female peers. To achieve this aim, we strengthened local capacity by including community health workers and care group women in a general training that focused on increasing knowledge related to the three intervention packages, and building facilitation and counseling skills. Based on reported behavior changes during TIPs, caregiver interest in the other intervention packages, and our intent to design an integrated infant and young child feeding and WASH intervention, we ultimately combined the three packages into a single behavior change strategy that would introduce one intervention package each month through neighbor women meetings and household counseling [66].

## Discussion

Due to its complex etiology, reductions in child stunting of the magnitude targeted for the Sustainable Development Goals requires the integration of multiple sectors [10]. When designed and implemented well, WASH strategies can reduce the burden of diarrheal diseases [22, 24, 67], though there has been little evidence of reducing asymptomatic chronic infections, and stunting reduction has not been demonstrated [22, 23]. To do so, interventions must achieve better adherence and sufficiently block relevant transmission pathways [68]. Health sector and mass media interventions can improve child feeding and nutrient intakes [69–72], and agriculture sector approaches may create an enabling food environment by improving diet quality, quantity, and safety [10, 11, 73]. Despite statistical significance, the impacts of direct nutritional programs to reduce stunting are relatively modest. Researchers attribute these modest impacts to several issues including limited multi-sectorial integration, limited application of contextually relevant and theoretically based behavior change theories and approaches [32, 41, 74–76], and the underutilization of theories of change in intervention design [28].

In reviewing the social/behavior change research literature, we noted that the experiences described in this paper aligned with several consistently cited recommendations for stunting prevention programs. They include the following:

1. Programs should be multi-sectorial to address multiple etiologies and include both community-based and family-focused approaches to reach not only mothers but also other influential family members such as fathers and mothers-in-law, peers, and policy makers [10, 54, 61, 74, 77–79].
2. Multi-sectoral interventions should be delivered in an integrated way that accounts for the lived experience, daily routines, and repeated behaviors of the caregivers who may be the sources of pathogen exposure and thus are expected to change behaviors [26, 80].
3. A deep understanding of context through formative research should inform intervention design and TOC development; the process for linking formative research findings to intervention components should be systematic and clearly articulated [28, 32, 50, 74, 81].
4. An evidence-based and theory-informed TOC should guide intervention design and be used throughout the program life cycle including implementation, monitoring, and evaluation [28, 29, 33, 39, 54, 82].
5. Intervention design should be grounded in *behavior change* theories; these theories should be explicitly articulated and utilized in the design of the theory of change [29, 54, 74, 83].
6. Interventions should be multi-level and multi-component integrating several relevant behavior change theories and including activities that target multiple (i.e., 2 or more) domains beyond knowledge generation [29, 54, 84, 85].
7. Interventions should engage not only phases of volitional behavior but maintenance as well, with potentially different but relevant theories and activities for each phase [86].
8. Pilot-testing should be conducted prior to larger scale implementation and testing to allow for refinement and revision of behavior change strategies and theories of change [47].
9. Stakeholders should be engaged in the development of the TOC and the design, implementation, and evaluation of interventions [28, 34].

To achieve alignment with these recommendations, our process adapted and integrated four methods—ethnographic formative research [50], an iterative and participatory process to develop the intervention’s theory of change [35], a systematically developed and evidence-based behavior change mapping exercise to identify intervention approaches and components [36], and behavioral micro-trials to design and pilot intervention components [47]. The formative research conducted for our work aimed to provide rich contextual information on the physical and social opportunity domains of our priority behaviors. It also provided deeper understanding of the motivations and capabilities that influenced behavior and highlighted the most appropriate community and family-focused activities and delivery platforms, including daily routines for integration. The findings of our formative work directly informed our iterative process to select priority behaviors, develop problem and solution trees for each behavior, and identify intervention packages and behavior change strategies most appropriate for the study context.

**Fig. 1:**
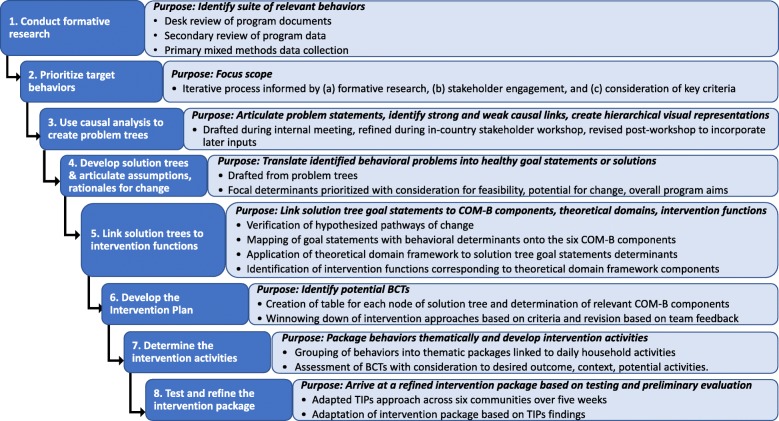
Eight-step process for intervention development and refinement. *BCTs* Behavior change techniques; *COM-B* Capability/opportunity/motivation behavioral model; *TIPs* Trials of improved practices

The BCW [36] served as a starting point for our work and a framework to identify relevant behavior change domains and the most appropriate techniques for achieving change of target behaviors in the study context (recommendation 5, above). For complex behaviors, such as those related to diet or WASH, the BCW approach recommends using the TDF to gain greater specificity in terms of the needed behavior changes. Collectively, this process is analogous to steps 3–5 in our process (Fig. 1). The application of the COM-B model, and subsequently the TDF to add greater specificity, mitigated the potential for an overly narrow focus that may arise in selecting and applying a single behavioral or behavior change theory to a complex behavior change problem [42, 58, 59]. Following detailed specification of the behaviors that required change, relevant and appropriate intervention functions were mapped to behavior change techniques to support development of intervention activities (i.e., steps 6 and 7, Fig. 1). While the BCW is a relatively new framework for intervention design, it has been used to characterize behavioral problems and develop interventions targeting both individual- and population-level health outcomes in the fields of mental health, nutrition and physical activity, smoking cessation, and improving provision of care by physicians and nurses [36]. While the bulk of BCW applications are in developed countries, examples exist from Thailand [87] and Kenya [88].

Many interventions in low- and middle-income countries targeting WASH and nutrition are criticized for their limited application of contextually grounded behavior change theories or inappropriate use of a single theory in intervention design [74]. Use of the BCW helps avoid these pitfalls through the use of context, most notably the social and environmental opportunities domain of the COM-B model, as a starting point and the integration of multiple behavior change theories to inform intervention design.

To be impactful, research suggests that interventions should focus intensively on a few key behaviors, integrate these into familiar routines, and appeal not only to early adopters, but also laggards and late adopters [26, 36, 74]. Through our intervention development process, we identified three intervention packages that were thematically coherent; each package focused on a series of behavior change activities that collectively served to motivate adoption of a set of thematically linked behaviors. The prioritized behaviors were grouped in ways that integrated into daily household routines, based either on performance at similar times of day or in similar locations. This routine-focused approach differs from the traditional siloing of WASH and nutrition messages based on behavioral outcomes. For example, messages around washing hands before eating were paired with mealtime behaviors, not other handwashing behaviors. Our approach allowed us to integrate multiple layers of intervention functions to reinforce behavior adoption and maintenance.

We used an adapted TIPs [47] approach to test the feasibility, acceptability, and potential effectiveness of the components of the intervention packages prior to larger scale testing. Through TIPs, we found that such thematic packaging enabled adoption of behaviors. However, the short duration and small sample of TIPs precluded us from examining behavior maintenance over a sustained period or assessing intervention outcomes.

TOCs, developed in the tradition of theory-driven evaluation, describe the underlying assumptions and hypothesized pathways by which programs achieve impacts—the how and the why of program impact. TOCs have multiple uses in the program life cycle but are often underutilized in program design and implementation [28]. Similarly, few programs describe in detail the process used to create their TOC. We adapted an existing framework and process for TOC development [35] and used a systematic behavioral mapping approach [36] to articulate rationales for our pathways to change (recommendation 4), although these methods could be applied to a range of different types of programs. TOCs can be used as “living” tools that adapt in response to lessons learned over the implementation period. In this project, TIPs served as a precursor to this adaptation process. As we have scaled the finalized intervention packages for larger implementation and testing, we have continued to revisit the TOC.

We recognized that stakeholder engagement in all aspects of our intervention development process would be crucial to ensure the intervention packages would be relevant and realistic to integrate into existing program context, sustainable and scalable. We sought a variety of relevant stakeholder perspectives that included topical (WASH, nutrition, behavior change), programmatic (country director, monitoring and evaluation experts, program managers), and contextual (local academic partner, local partner organizations) expertise and experiences. Stakeholders were engaged through participatory workshops, monthly conference calls, and feedback integration at each stage of the process. While some activities were inherently collaborative in nature, others more closely resembled a system of product review, provision of feedback, and subsequent revision. Applying stakeholder feedback to the development of interventions in other areas would likely involve other differences in how stakeholders are engaged.

## Strengths, limitations, and challenges

Our intervention design process had several limitations worth noting. First, the process of finalizing the behaviors to focus on was iterative, which is both a strength and a limitation. As a strength, it allowed for the voices of relevant stakeholders to be taken into consideration. But as a limitation, it served as a “constantly moving target”; as behaviors were continually refined, so too did the need to refine the foundational work (i.e., the problem and solution trees), the intervention functions, and even the intervention plan.

Second, we deliberately developed this intervention within the context of the Care Group model. Though it enhanced the potential for scale, this decision limited the nature of the intervention activities as this model targets individual- and/or community-level behaviors (versus policy or physical structures). Adherence to these criteria limited our ability to fully elaborate interventions based on our application of the BCW approach, especially as they pertain to creating an enabling environment for change. For example, environmental opportunities that influence diet practices, such as food accessibility and affordability, could be enhanced through policy levers including for example, subsidies for high-quality foods (i.e., a fiscal measure in BCW vernacular) or enhanced agriculture extension (i.e., service provision). Large scale mass media efforts can effectively shift social norms and behaviors [32, 63, 77–79] and may be useful for inculcating and reinforcing handwashing and feces disposal. Similarly, platforms that use the education sector to target school-aged children can shift beliefs, attitudes, and practices in the home [89]; however, these were outside the scope of the THRIVE II delivery platform.

Third, it is unclear the extent to which the intervention that was developed is generalizable beyond western Kenya. On the one hand, the extensive formative research ensures that the intervention is sensitive to context, but in doing so, it may be less applicable in contexts outside of this setting. Future work will hopefully allow us to test and modify the approach; the process laid out in this manuscript provides a roadmap. Finally, examining the approaches as a complete set of intervention packages masks whether there are subcomponents that are more or less effective at generating behavioral change. A dismantling research design in which individual intervention components are tested separately (or in subsets) would be needed to more fully answer this question.

## Conclusions

We have outlined our approach to developing a theory- and evidence-based intervention that integrated a set of WASH and nutrition behaviors to target growth shortfalls among young children in western Kenya. Interventions, specifically in the WASH and nutrition sectors are not typically developed in such a prescriptive way, yielding poor sustainability and sub-optimal health gains. An approach that equally values peer-reviewed evidence and stakeholder inputs, and that adheres to a structured and deliberate theory-based process may provide the best opportunity to achieve interventions that result in sustained behavior change at scale. We hope that in laying out this step-by-step process, others may use and modify our approach to support rigorous program design in a range of other areas of needed behavior change.

## Data Availability

The datasets used and/or analyzed during the current study are available from the corresponding author on reasonable request.

## Abbreviations

BCW: Behavior change wheel
COM-B: Capability, opportunity, motivation-behavior
TDF: Theoretical domain framework
TIP: Trials of improved practice
TOC: Theory of change
WASH: Water, sanitation, and hygiene
